# Book Reviews

**Published:** 1889-03

**Authors:** 


### BOOK REVIEWS.

Albuminuria. By Thomas Grainger Stewart, M.D., Fellow of the Royal College of Physicians, Edinburg; Hon. Fellow of King and Queen's College of Physicians, Ireland; Physician in Ordinary to Her Majesty the Queen for Scotland. Pp. xi. and 250. New York: William Wood & Co. Chicago: W. T. Keener.

This book comprises a series of clinical lectures delivered during the past two years at the University of Edinburgh. They embody the views which the author at present entertains regarding the clinical questions discussed in his well known work upon "Bright's Diseases of the Kidneys"—the last edition of which appeared in 1871.

The first chapter is devoted to the consideration of the various proteids found in the urine, together with their various tests, qualitative and quantitative.

Of the more recent tests for albumin in the urine, in point of delicacy the author gives the preference to picric acid. But he wisely remarks "delicacy is not the only quality required of a test. Indeed, it may be too delicate for clinical purposes."

In the second chapter Professor Stewart considers the "Incidence of Albuminuria among the Presumably Healthy." From the number of observations, and the care with which they have been conducted, some very important conclusions have been reached, more especially with regard to the relations of albuminuria to life assurance.

Systematic observation of the urine of 505 presumably healthy individuals was made. Some of the more important deductions reached through these researches are as follows:

(a). "That albuminuria is much more common among presumably healthy people than was formerly supposed, being demonstrable by delicate tests in nearly one-third of those examined."

(b). "That the existence of albuminuria is not of itself sufficient ground for the rejection of a proposal for life insurance."

(c). "That traces of albumin are not unfrequently present in the urine passed during the first days of life."

(d). "That excepting as above shown, the frequency of albuminuria increases as life advances, being rare in children and young adults, and common in men at or above sixty years of age."

In the third chapter the author considers the "Incidence of Albuminuria among the Sick" and from a large number of observations concludes that "cases of Bright's disease do not account for one-half of the cases of albuminuria."

The "Theory of Albuminuria" is ably discussed in chapter four." The author lays much stress upon inflammatory changes in the epithelium of the renal tubules as a frequent cause. He believes, however, that increased blood-pressure, as well as some conditions of the blood, favors its occurrence.

The succeeding five chapters are devoted to the consideration of the various organic diseases of the kidneys which are accompanied by albuminuria. The author's views upon these subjects are already very widely known through his excellent work on Bright's diseases. We may add, however, that the present matter is brought very fully up to date.

In the eleventh chapter the author discusses the important question of "functional albuminuria" in its various forms. In regard to the prognosis of these cases, the author believes that "Paroxysmal Albuminuria" has a slight tendency to culminate in renal disease. In "Dietetic Albuminuria" and "Albuminuria from Muscular Exertion" there is less tendency to such sequel. In "Simple Persistent Albuminuria" the prognosis is least hopeful, but in the author's experience "so considerable a proportion of cases have gone on for long periods" without culminating in organic renal disease that he is confident an unfavorable termination of these cases must be rare.

The excretion of the normal proportion of solids by the kidneys, and the absence of vascular changes, the author considers—we believe very wisely—to be the most weighty evidences against the existence of serious damage to the organs.

The last two chapters of the book deal with the management of albuminuria, including the diet. In the author's experience "raw egg albumin" when introduced into the stomach induces albuminuria, "that the albumin is always small in quantity," that "it disappears when ordinary diet is resumed," and that it is not egg albumin but serum albumin which is discharged. With regard to the use of cheese the author concludes that "when eaten in reasonable amount it has little or no effect in producing albuminuria in healthy people."

In organic renal disease the author does not expect much from the use of medicines save to combat complications. In albuminuria due to cardiac disease, however, "most strikingly good effects follow the use of appropriate medicines" such as digitalis, strophanthus, caffein, convallaria majolis, etc. In "functional albuminuria" the author has found the best results from the use of arsenic, chloride of ammonium and iron.

Professor Stewart's book may be looked upon as a valuable contribution to the literature of albuminuria. His lengthy experience as a clinical teacher has enabled him to present the matter in a most practical form, and his industrious tabulation of statistics of the large number of cases observed greatly facilitates the study of the subject by the student and practitioner, while his well-known reputation as an author on renal diseases lends very great weight to the conclusions which he so clearly and pleasantly presents. We heartily recommend the work to all those who wisk to make themselves acquainted with the most recent advances on the subject of albuminuria. C. W. P.

---

Electricity in the Diseases of Women, with Special Reference to the Employment of Strong Currents. By G. Betton Massey, M.D. Pp. viii and 210. 12mo. Philadelphia: F. A. Davis. 1889. Price, $1.50 net.

There will always be found some practitioners in any department of medicine who are so successful in the use of their own methods, that they are eminently satisfied with them and are unwilling to employ any others. And thus there are gynæcologists who succeed better with other agents and condemn electricity as the resource of charlatans or unskilful operators. But the world moves, and from each of the fashions of its day something is left which is a valuable contribution to the resources of medicine, and those who will carefully read what the author of this little book on Electricity in Diseases of Women has to say, will realize that that agent has in some particulars passed its experimental stage and is capable of accurate and satisfactory application.

The book has many good points. Its descriptions of apparatus and methods are perfectiy clear and accurate, and will enable any physician to use even strong currents intelligently and safely. For his own current controller, an extremely simple and efficient instrument, he is entitled to the thanks of the profession.

The limitations as well as the uses of the agent as they are at present known are clearly stated. The author's style is exceptionally clear and terse, and the arrangement of the subject matter in the book is excellent. The illustrations are well chosen. And lastly, although amply covering the ground the book is small. E. W.

